# Prevalence and risk factors of HIV infection among people who inject drugs in Cambodia: findings from a national survey

**DOI:** 10.1186/s13011-019-0232-3

**Published:** 2019-10-17

**Authors:** Gitau Mburu, Pheak Chhoun, Navy Chann, Sovannary Tuot, Phalkun Mun, Siyan Yi

**Affiliations:** 1 Division of Health Research, Lancaster University, Lancaster, UK; 2 Centre for Global Health Policy, University of Sussex, Brighton, UK; 3 KHANA Center for Population Health Research, Phnom Penh, Cambodia; 4 National Center for HIV/AIDS, Dermatology and STD, Phnom Penh, Cambodia; 5 Center for Global Health Research, Touro University California, Vallejo, USA; 6 Saw Swee Hock School of Public Health, National University of Singapore and National University Health System, Singapore, Singapore

**Keywords:** HIV risk, Injecting drug use, Harm reduction, National survey, Prevalence, Cambodia

## Abstract

**Background:**

Globally, people who inject drugs (PWID) continue to be among the most vulnerable populations to acquire infection of human immunodeficiency virus (HIV). The most recent national survey found that nearly a quarter of PWID in Cambodia were infected with HIV in 2012. The aim of this study is to estimate the current prevalence of and factors associated with HIV infection among PWID in Cambodia.

**Methods:**

This national integrated biological and behavioral survey was conducted from June to December 2017. Participants were recruited from the capital city and 11 major provinces using the respondent driven sampling method. Face-to-face interviews were conducted using a structured questionnaire, and blood samples were collected for HIV, syphilis, and hepatitis C virus (HCV) testing. Multiple logistic regression analysis was conducted to identify risk factors for HIV infection. All analyses were estimated with sampling weights that corrected for non-response and sample design.

**Results:**

A total of 310 PWID participated in the study, and the mean age was 31.8 years (SD = 7.8). The prevalence of HIV was 15.2%. More than half (57.4%) of the HIV-positive participants were co-infected with HCV, and 44.7% were not aware of their HIV status prior to this study. After adjustment for other covariates, HIV infection remained positively associated with being female (AOR = 1.88, 95% CI = 1.03–4.04), being in the older age group of ≥35 (AOR = 2.99, 95% CI 1.33–9.22), being widowed, divorced or separated (AOR = 2.57, 95% CI = 1.04–6.67), living on the streets (AOR = 2.86, 95% CI 1.24–4.37), and HCV infection (AOR = 3.89, 95% CI 1.86–1.15). The HIV infection remained negatively associated with having higher level of formal education of ≥10 years (AOR = 0.44, 95% CI 0.13–0.83) and higher average income of ≥US$200 per month (AOR = 0.20, 95% CI = 0.05–0.74).

**Conclusions:**

The prevalence of HIV among PWID in Cambodia remains high, but is reducing compared with the 24.8% reported in the 2012 national survey. Findings from this study provide critical information for tailoring interventions based on identified vulnerabilities and risk factors for HIV. Our findings underline the importance of socio-structural factors in HIV epidemiology among PWID in Cambodia, which require mitigation.

## Background

In Cambodia, people who inject drugs (PWID) are a priority group for HIV prevention, as they comprise an important key population at risk of human immunodeficiency virus (HIV) infection [1, 2]. Previous studies in the country have characterized the HIV epidemic among PWID. A national Integrated Biological and Behavioral Survey (IBBS) conducted in 2012 reported that the prevalence of HIV among PWID in Cambodia was 24.8% [3]. The vast majority of Cambodian PWID are male, reside in urban areas, and mostly inject heroin [3, 4]. Documented risk factors for HIV acquisition among PWID in Cambodia include needle/syringe sharing [3, 5].

To respond to the needs of PWID, intensive HIV prevention interventions have been implemented throughout the country as part of the Boosted Continuum of Prevention, Care, and Treatment (B-CoPCT) strategy, which was initiated in 2012 [1, 2]. B-CoPCT strategy aims to achieve the “Three Zeros” (i.e. zero new HIV infection, zero discrimination, and zero AIDS-related deaths) by 2020 [2, 6, 7]. B-CoPCT interventions for PWID include needle and syringe exchange programs, condom promotion, peer education, community-based outreach, medically assisted therapy, and peer-led HIV voluntary confidential counseling and testing, which are provided via both governmental and non-governmental organizations (NGOs) [3, 4]. As a result of these efforts, Cambodia has made tremendous progress in reducing HIV prevalence and incidence [2], and is one of the few countries in the world that are close to achieving the 90–90-90 global targets by 2020 [8].

As the HIV epidemic abates, focus has shifted to an HIV case detection model that ensures linkage to care and treatment for all detected infections [2, 4]. As in other settings [9–11], there is an emerging consensus that eliminating the HIV epidemic in Cambodia requires granulating the HIV risk factors and addressing them at the local level, for each of the key populations at risk of HIV [1, 4]. Therefore, a critical part of ongoing HIV prevention is understanding emerging risk profiles and ensuring that HIV programs respond to different strata of PWID. It is in this context that the government has committed to strengthen the strategic information related to PWID, including separating them from the general category of people who use drugs (PWUD) [1]. This separation is essential to ensure that provision of clean needles, syringes, and medical assisted therapy is optimized among people who specifically inject drugs as recommended by the World Health Organization [12]. Lumping injectors and non-injectors may fail to distinguish varying levels of risk between people who inject and those who consume drugs by other modes [13].

To achieve Cambodia’s national HIV prevention goals, routine behavioral surveillance of key populations is routinely conducted to characterize national trends in the epidemic, and strategic information informs the development of effective HIV interventions. This paper focusses on PWID and aims to report: (1) an estimate of HIV prevalence; (2) factors associated with HIV infection; and (3) potential ways in which programs can be adjusted to enhance HIV prevention among this key population in Cambodia.

## Methods

### Study design, sites, and participants

A cross-sectional survey was conducted from June to December 2017. Data were collected from participants in the capital city of Phnom Penh and 11 major provinces, which were purposively selected following a feasibility assessment. These 12 study sites contained 21 operational districts with high-burden of drug use and HIV. PWID were defined as people who have injected any type of illicit drugs, as specified by the Cambodian Law on Control of Drugs, in the past 12 months [14].

### Inclusion and exclusion criteria

Individuals would be included in the study if they: (1) were 18 years or older; (2) presented a valid study recruitment coupon; (3) injected any illicit drug in the past 12 months; and (4) were willing and able to provide informed consent for study participation. To prevent multiple participation, participants were excluded if they had already participated in this survey elsewhere in the country.

### Sample size calculation

The sample size calculation was based on an estimated PWID population size of 1300 [3] and an assumption of a 20% decrease in the HIV prevalence since the 2012 IBBS. Using a margin of error of 1.5%, a confidence interval of 95%, a 90% response rate and a design effect of 1.5, the minimum sample size required for this study was 290. Roughly 25.0% of the estimated 1068 PWID in Phnom Penh in 2016 [15] were recruited, assuming that, based on programs reports, there would be no PWID in sites outside the capital city. However, PWID found in any sites would be included in the study.

### Recruitment

The Respondent Driven Sampling (RDS) method was used to recruit study participants, and the Strengthening the Reporting of Observational Studies in Epidemiology for RDS Studies (STROBE-RDS) statement was followed [16]. RDS is a network-based method to recruit participants from hidden populations such as people who use drugs, commercial sex workers, and men who have sex with men [17], and is frequently used to estimate characteristics of hard-to-reach groups, such as the HIV prevalence [16].

The RDS was implemented in five stages. First, four eligible PWID who were well-connected to other PWID in each location were selected as seeds to recruit other PWID in their network. Second, each seed was given a Personal Identification Number (PIN) and enrolled as a participant. Third, each seed received three coupons and was asked to refer three additional PWID. Seeds received US$2 for each successful recruit, while each recruit received a gift costing approximately US$5 to compensate for their time and transport. Each seed was expected to extend up to 3 to 6 “recruitment waves” in each site. If the initial seeds did not recruit participants or if enrollment has been halted because all recruitment chains have “dried up” (i.e. stopped recruiting), additional seeds would be selected based on the above criteria. All recruits were provided the same opportunity as seeds to recruit other PWID.

### Data collection training

A data collection team was provided with a three-day training on the study protocol and the process of data collection to ensure that the team members were thoroughly familiar with the study. The training workshop covered skills such as interview techniques as well as participant confidentiality and privacy projection. It also provided the team with an opportunity to practice questionnaire administration and pretest the tools. Regular daily review sessions with interviewers were conducted during the data collection to review progress and communicate and resolve any issues encountered.

### Data collection procedures

#### Biological data collection

HIV and syphilis screening was conducted with capillary blood by a laboratory technician using SD Bioline HIV/Syphilis Duo Test (Standard Diagnostic Inc., Korea). A non HIV-reactive result establishes that an individual is not HIV-infected. HIV reactive results were confirmed using HIV 1/2 STAT-PAK® Assay (Chembio Diagnostic 127 Systems Inc., New York). HCV antibody testing was performed using capillary blood with HCV Oraquick (OraSure Technologies, Inc., Bethlehem). Participants received their results verbally in a post-test counseling session after the interview. All newly identified HIV and syphilis cases were linked to a local NGO in the area for further management according to national guidelines. HCV-positive cases were referred to Médecins Sans Frontières for care and treatment support.

#### Questionnaire development and measures

Standardized and validated tools were adapted from previous studies among HIV key populations in Cambodia and the most recent Cambodia Demographic and Health Survey [3, 18–21]. The structured questionnaire was initially developed in English and then translated into Khmer, the national language of Cambodia. It was then back-translated into English by another translator to ensure that the “content and spirit” of every original item were maintained. A consultative meeting was held with representatives of key stakeholders working on HIV and harm reduction and community people to review the study protocol and tools. A pilot study was conducted with 10 PWID in Phnom Penh, who were later excluded from the main study.

Socio-demographic characteristics included type of community (urban or rural), age (continuous), gender (male or female), years of formal education attained (continuous), average income in the past six months (continuous), living situation (homeless, with family, own dwelling, with friends, or other), employment status (unemployed, entertainment worker, office worker, laborer/farmer, or other), ethnicity (Khmer, Vietnamese, or other), and current marital status (married, never married, or widowed/divorced/separated).

Regarding drug use, we collected information on types of illicit drugs and frequency of use in the past three months. These include the use of drugs (yes or no) and type of drugs used (heroin, Yama/methamphetamine, ecstasy or inhalants) in the last three months. To assess risky injecting behaviors, PWID were asked about consistent use of new needles/syringes and sharing of needles with other PWID. Alcohol use was assessed by asking participants if they consumed alcohol ≥3 times per week and whether they binge drank ≥3times per week. To measure HIV risks, participants were asked about their sexual behaviors in the past three months including number of partners and condom use with commercial (defined as a partner with whom the participant had sex in exchange for money or goods) and non-commercial partners in the past three months. We also collected information regarding STI symptoms and exposure to community-based HIV, harm reduction, and other related services in the past six months.

### Statistical analyses

All analyses were estimated with sampling weights that corrected for nonresponse and sample design [22]. The prevalence of HIV was calculated by dividing the total number of HIV-positive participants with the total number of participants tested. Characteristics and behavioral variables of HIV-positive participants were compared to those of HIV-negative participants using Chi-square test (or Fisher’s exact test for an expected cell value of ≤5) for categorical variables and Student’s *t*-test or Mann-Whitney U test for continuous variables. Age, level of education, and income were transformed into categorical variables. To identify risk factors for HIV infection, variables associated with HIV infection at a significance level of *p* < 0.05 in bivariate analyses were simultaneously included in a multiple logistic regression model. Age, gender, level of education, and income were included in the model regardless of the significance level in bivariate analyses. Backward stepwise selection method was used to eliminate variables with the highest *p*-value one-by-one from the model. Adjusted odds ratios (AOR) and 95% confidence intervals (CI) were calculated. STATA Version 12.0 for Windows (Stata Corp, TX, USA) was used for the analyses.

## Results

### Prevalence of HIV

This study included 310 PWID with a mean age of 31.8 years (SD = 7.8). Forty-seven participants (15.2%) tested positive for HIV (95% CI = 4.6–7.1%). More than half (57.4%) of the HIV-positive individuals were co-infected with HCV. The majority of the HIV-positive cases (95.7%) were found in Phnom Penh, and 44.7% were not aware of their HIV status prior to the study. Of the 26 cases who were aware of their HIV status, 84.0% were on ART.

### Socio-demographic characteristics

The majority (70.3%) of the participants lived in Phnom Penh. As shown in Tables 1, 91.6% resided in urban areas; 73.2% were male; 40.6% were married; and 56.1% had attained only primary education. While 39.4% reported living with their family or relatives, 27.1% were living on the streets. The most common job was a laborer or farmer (37.4%), and 12.6% were unemployed. The majority (78.6%) reported an average monthly income in the past six months of <US$200. A significantly higher proportion of HIV-positive participants were in the age group of ≥35 (57.4% vs. 35.5%, *p* < 0.001); widowed, divorced, or separated (34.0% vs. 16.3%, *p* = 0.008); had completed only primary formal education (72.3% vs. 53.2%, *p* = 0.008); lived on the streets (44.7% vs. 24.0%, *p* = 0.003); and had an average monthly income of <US$100 (46.8% vs. 34.4%, *p* = 0.009) compared to the HIV-negative group.

**Table 1:** Socio-demographic characteristics of HIV-positive and HIV-negative PWID

	Total (*n* = 310)	HIV test result		
		Positive (*n* = 47)	Negative (*n* = 263)	
	*n* (%)	*n* (%)	*n* (%)	*P*-value^*^
Living in an urban community	284 (91.6)	46 (97.9)	238 (90.5)	0.15
Female gender	83 (26.8)	18 (38.3)	65 (24.7)	0.07
Age group				0.001
18–24	59 (19.1)	1 (2.1)	58 (22.1)	
25–34	130 (42.1)	19 (40.4)	111 (42.4)	
≥ 35	120 (38.8)	27 (57.4)	93 (35.5)	
Ethnicity				0.23
Khmer	244 (79.0)	34 (72.3)	210 (80.2)	
Vietnam	65 (21.0)	13 (27.7)	52 (19.8)	
Current marital status				0.008
Never married	125 (40.3)	12 (25.5)	113 (43.0)	
Married	126 (40.6)	19 (40.4)	107 (40.7)	
Widowed/divorced/separated	59 (19.0)	16 (34.0)	43 (16.3)	
Level of formal education completed				0.03
Primary (0–6 years)	174 (56.1)	34 (72.3)	140 (53.2)	
Secondary school (7–9 years)	75 (24.2)	9 (19.1)	66 (25.1)	
High school or higher (≥10 years)	61 (19.7)	4 (8.5)	57 (21.7)	
Living arrangement				0.003
In the street (homeless)	84 (27.1)	21 (44.7)	63 (24.0)	
With family or relatives	122 (39.4)	11 (23.4)	111 (42.2)	
In own dwelling	60 (19.4)	5 (10.6)	55 (20.9)	
With friends	15 (4.8)	5 (10.6)	10 (3.8)	
Other	29 (9.4)	5 (10.6)	24 (9.1)	
Main occupation				0.74
Unemployed	39 (12.6)	5 (10.6)	34 (12.9)	
Entertainment worker	32 (10.3)	3 (6.4)	29 (11.0)	
Office worker	79 (25.5)	15 (31.9)	64 (24.3)	
Laborer/farmer	116 (37.4)	17 (36.2)	99 (37.6)	
Other	44 (14.2)	7 (14.9)	37 (14.1)	
Average monthly income in the past 6 months (US$)				0.009
< 100	112 (36.2)	22 (46.8)	90 (34.4)	
100–199	131 (42.4)	22 (46.8)	109 (41.6)	
≥ 200	66 (21.4)	3 (6.4)	63 (24.0)	

*Abbreviations: HIV* human immunodeficiency virus*, PWID* people who inject drugs

^*^Chi-square (or Fisher’s exact test when a cell count was smaller than 5) was used

### Substance use

Table 2 shows that heroin was the most commonly used drug in the past three months (60.4%), followed by Yama/ice (common name for methamphetamine in Cambodia) (24.2%). About two-thirds (64.8%) reported that they always used new syringes/needles for drug injection in the past three months, while the remaining 23.5% reported using needles or syringes that had been used by someone else over the same period. Alcohol use was also common with 29.7% reporting alcohol drinking at least three times per week; of these, 47.4% reported binge drinking (drinking at least five units of alcoholic drinks on a typical day) at least three days per week in the past three months. A significantly lower proportion of HIV-positive participants reported alcohol drinking ≥3 times per week in the past three months (8.5% vs. 33.5%, *p* = 0.001).

**Table 2:** Substance use among HIV-positive and HIV-negative PWID

	Total (*n* = 310)	HIV test result		
		Positive (*n* = 47)	Negative (*n* = 263)	
	*n* (%)	*n* (%)	*n* (%)	*P*-value^*^
Used any drugs in the past 3 months	271 (88.0)	43 (91.5)	228 (87.4)	0.63
Type of drugs most commonly used in the past 3 months				
Heroin	165 (60.4)	29 (67.4)	136 (59.1)	0.06
Yama/ice (methamphetamine)	66 (24.2)	10 (23.3)	56 (24.3)	0.88
Ecstasy	11 (4.0)	0 (0.0)	11 (4.8)	0.22
Inhalants	5 (1.8)	0 (0.0)	5 (2.2)	1.00
Always used new syringes/needles	118 (64.8)	16 (64.0)	102 (65.0)	0.93
Used needles/syringes used by someone else in the past 3 months	43 (23.5)	4 (16.0)	39 (24.7)	0.38
Alcohol drinking ≥3 times per week	92 (29.7)	4 (8.5)	88 (33.5)	0.001
Binge drinking ≥3times per week (*n* = 190)	90 (47.4)	6 (35.3)	84 (48.6)	0.30

*HIV* human immunodeficiency virus*, IQR* interquartile range, *PWID* people who inject drugs

^*^Chi-square (or Fisher’s exact test when a cell count was smaller than 5) was used for categorical variables and Mann-Whitney U test for continuous variables

### Sexual behaviors

As shown in Tables 3, 17.5% reported always using condoms, while 45.1% reported having sex when a partner was intoxicated in the past three months. Of those who reported having sex with partners not in exchange for money or gifts (*n* = 117), 8.5% reported always using condoms with the non-commercial partners in the past three months. Of the total respondents, 22.6% reported having sex in exchange for money or gifts in the past three months; of whom, 27.3% reported always using condoms with the commercial partners in the past three months. No significant difference was found in comparison of sexual behaviors in HIV-positive and HIV-negative group.

**Table 3:** Sexual behaviors and perceived HIV risk among HIV-positive and HIV-negative PWID

	Total (*n* = 310)	HIV test result		
		Positive (*n* = 47)	Negative (*n* = 263)	
	*n* (%)	*n* (%)	*n* (%)	*P*-value^*^
Median number of sex partners (IQR)	1.0 (0.0–2.0)	1.0 (0.0–1.0)	1.0 (0.0–2.0)	0.50
Always used condom with any partner	34 (17.5)	6 (21.4)	28 (16.9)	0.56
Had sex when a partner was intoxicated	87 (45.1)	9 (32.1)	78 (47.3)	0.14
Had sex with partners not in exchange for money or gift	117 (60.3)	19 (67.9)	98 (59.0)	0.38
Always used condom with partners not in exchange for money or gift	10 (8.5)	3 (15.8)	7 (7.1)	0.21
Had sex in exchange for money or gifts	44 (22.6)	7 (25.0)	37 (22.2)	0.74
Always used condom with partners in exchange for money or gift	12 (27.3)	2 (28.6)	10 (27.0)	0.93

*HIV* human immunodeficiency virus*, IQR* interquartile range, *PWID* people who inject drugs

^*^Chi-square (or Fisher’s exact test when a cell count was smaller than 5) was used for categorical variables and Mann-Whitney U test for continuous variables

### Hepatitis C and STIs

As shown in Tables 4, 28.1% of the participants were tested positive for HCV and 5.2% for syphilis, and 28.2% reported having had at least one STI symptom in the past 12 months. The most commonly reported symptoms included abnormal urethral discharge (65.9%), followed by swelling around the genital area (26.1%) and having cuts or ulcerations around the genital area (25.0%). Compared to HIV-negative group, a significantly higher proportion of HIV-positive participants were tested positive for HCV (57.4% vs. 22.8%, *p* < 0.001).

**Table 4:** Comparisons of STI symptoms among HIV-positive and HIV-negative PWID

	Total (*n* = 310)	HIV test result		
		Positive (*n* = 47)	Negative (*n* = 263)	
	*n* (%)	*n* (%)	*n* (%)	*P*-value^*^
Tested positive for hepatitis C	87 (28.1)	27 (57.4)	60 (22.8)	< 0.001
Tested positive for syphilis	16 (5.2)	3 (6.4)	13 (4.9)	0.72
Had any STI symptoms	87 (28.2)	12 (25.5)	75 (28.6)	0.66
Cuts or sores around the genitals	22 (25.0)	1 (8.3)	21 (27.6)	0.28
Swelling around the genitals	23 (26.1)	1 (8.3)	22 (28.9)	0.17
Abnormal urethral discharge	58 (65.9)	9 (75.0)	49 (64.5)	0.74
Symptoms around the anus	12 (13.6)	2 (16.7)	10 (13.2)	0.67
Symptoms in the mouth or throat	10 (11.4)	0 (0.0)	10 (13.2)	0.35
Received treatment for most recent STI	69 (79.3)	11 (91.7)	58 (77.3)	0.26

*HIV* human immunodeficiency virus*, PWID* people who inject drugs, *STI* sexually transmitted infections

^*^Chi-square or Fisher’s exact test was used as appropriate

#### Access to community-based HIV services

Table 5 shows that 68.4% of the study participants reported having received some form of community-based HIV services in the past six months. The services included condom and lubricant distribution (69.9%), HIV testing (60.8%), HIV education (47.9%), needle and syringe distribution (49.0%), methadone maintenance therapy (41.9%), drop-in services (21.7%), and HCV testing (20.3%). The proportion of participants who reported having received overall community-based HIV services (80.9% vs. 66.2%, *p* = 0.04) and methadone maintenance therapy (72.3% vs. 36.9%, *p* < 0.001) in the past six months was significantly higher among HIV-positive participants compared to that among HIV-negative group.

**Table 5:** Access to community-based HIV services among HIV-positive and HIV-negative PWID

	Total (*n* = 310)	HIV test result		
		Positive (*n* = 47)	Negative (*n* = 263)	
	*n* (%)	*n* (%)	*n* (%)	*P*-value^*^
Received community-based HIV services	212 (68.4)	38 (80.9)	174 (66.2)	0.04
HIV education services	68 (47.9)	15 (57.7)	53 (45.7)	0.27
Condom and lube distribution	100 (69.9)	22 (84.6)	78 (66.7)	0.10
Needle and syringe distribution	70 (49.0)	15 (57.7)	55 (47.0)	0.32
HIV/STI testing services	87 (60.8)	16 (61.5)	71 (60.7)	0.94
Hepatitis C testing services	63 (20.3)	15 (31.9)	48 (18.3)	0.03
Methadone maintenance therapy	130 (41.9)	34 (72.3)	96 (36.5)	< 0.001

*HCV* hepatitis C virus, *HIV* human immunodeficiency virus*, PWID* people who inject drugs, *STI* sexually transmitted infections

^*^Chi-square or Fisher’s exact test was used as appropriate

### Factors associated with HIV infection

Factors associated with HIV infection among PWID in this study are shown in Table 6. After adjustment for other covariates, HIV infection remained positively associated with being female (AOR = 1.88, 95% CI = 1.03–4.04), being in the older age group of ≥35 (AOR = 2.99, 95% CI 1.33–9.22), being widowed, divorced or separated (AOR = 2.57, 95% CI = 1.04–6.67), living on the streets (AOR = 2.86, 95% CI 1.24–4.37), and HCV infection (AOR = 3.89, 95% CI 1.86–1.15). The HIV infection remained negatively associated with having higher level of formal education of ≥10 years (AOR = 0.44, 95% CI 0.13–0.83) and higher average income in the past six months of ≥ US$200 per month (AOR = 0.20, 95% CI = 0.05–0.74).

**Table 6:** Factors associated with HIV infection among PWID in multivariate logistic regression model

Variables in the final model*	AOR (95% CI)
Gender	
Male	Reference
Female	1.88 (1.03–4.04)
Age group	
< 25	Reference
25–34	1.44 (0.66–4.47)
≥ 35	2.99 (1.33–9.22)
Current marital status	
Never married	Reference
Married	0.96 (0.39–2.34)
Widowed/divorced/separated	2.57 (1.04–6.67)
Level of formal education completed	
Primary (0–6 years)	Reference
Secondary school (7–9 years)	0.85 (0.35–2.05)
High school or higher (≥10 years)	0.44 (0.13–0.83)
Average monthly income in the past 6 months (US$)	
< 100	Reference
100–199	0.94 (0.45–1.94)
≥ 200	0.20 (0.05–0.74)
Living arrangement	
With family/relatives	Reference
On the streets (homeless)	2.86 (1.24–4.37)
In own dwelling	1.19 (0.53–7.95)
With friends	1.48 (0.62–9.74)
HCV test result	
Negative	Reference
Positive	3.89 (1.86–8.15)

*AOR* adjusted odds ratio*, CI* confidence interval*, HCV* hepatitis C virus, *HIV* human immunodeficiency virus*, PWID* people who inject drugs

^*^Age, gender, marital status, education level, entertainment venue, and variables associated with HIV infection in the bivariate analyses at a level of *p* < 0.05 were simultaneously included in the model

## Discussion

A previous national IBBS conducted in year 2012 among PWID found an HIV prevalence of 24.8% [3, 5]. This 2017 IBBS demonstrates a shift in this prevalence showing that the current prevalence had reduced to 15.2%. At the most basic level, this new prevalence is still about 25 times higher than the estimated 0.6% among the general adult population aged 15–45 in 2016 [23], and suggests the need to continue focussing on this population, alongside other key populations. Furthermore, our results show that 57.4% of HIV-infected PWID were co-infected with HCV. This study has also identified a number of risk factors of HIV infection including female gender, older age, low level of formal education, low income, homelessness, being widowed/divorced/separated, and having HCV infection.

The finding that being female almost doubled the odds of HIV infection is in keeping with previous studies from other global contexts showing that women are more vulnerable to HIV [24, 25]. Female gender was also identified as a predictor of HIV infection in the 2012 IBBS in Cambodia that included both PWUD and PWID [3, 5]. While this vulnerability could be due to injection risks, it is possible that this risk is overlaid with sexual risks, such as multiple sexual partners and unprotected sex, as is the case in other contexts [26, 27]. In Cambodia, a better understanding of the profiles of female PWID, including whether they are engaged in sex work to cater for their drug use, and whether their partners inject drugs, could provide further information on risk profiles and assist in HIV prevention programming.

The finding of older age being associated with higher prevalence is similar to findings among other key populations in Cambodia [7, 28]. It is plausible that, as PWID continue to inject, they have more opportunities to acquire HIV [29]. Older age and longer duration of drug use was also identified as a predictor of HIV infection among PWUD and PWID in the 2012 IBBS in Cambodia [3, 5].

Our results show that being homeless was associated with HIV infection. Other studies have shown that homelessness and mobile lifestyles are predictive of both the likelihood to inject [30], as well as poor uptake of harm reduction interventions among PWID [31]. Even in high resource-settings such as Canada and Australia, injecting drugs is frequently associated with both unemployment and homelessness [30, 32]. In turn, homelessness is likely to increase the risk of HIV infection [33, 34] by acting as a structural barrier to accessing harm reduction interventions [31, 35]. Homelessness encourages sharing and other risk taking among PWID [35]. On a practical level, this finding provides a useful way in which to categorize profiles of HIV risk among PWID and suggests that outreach to homeless PWID will be essential in HIV case finding and prevention. This is particularly important given that 27.1% of the sampled PWID were living on the streets.

Because unsafe injection is a common risk factor for parenteral acquisition of both HIV and HCV [36, 37], it is not surprising that a significant proportion of PWID in this study were co-infected with both of these viral infections. Among adults living with HIV in Cambodia, HIV/HCV co-infection rates between 5.3 and 10.5% have been reported [38–40]. However, these previous studies included a limited number of PWID, as compared to the heterosexual general population. As such, the higher prevalence of co-infection in this IBBS can be explained by the fact that our sample was exclusively composed of PWID.

In our study, having completed at least high school level of formal education almost halved the odds of having HIV. This finding is consistent with previous studies in Cambodia that have demonstrated that higher levels of education are protective against HIV infection [7, 41]. The finding that lower income is predictive of HIV infection could be related to the impact of structural economics on injection risks. Economic considerations and being in socially deprived situations may indirectly influence risk taking behaviors such as needle sharing [42]. This might explain why widowed, divorced or separated PWID had a higher risk of HIV, although this may also result from sexual acquisition of HIV from their partners, whose HIV status was not enquired or documented.

### Policy implications

Our findings show the need to focus on macro-social and structural factors that determine HIV acquisition including homelessness, gender, and economic power. Therefore, such macro level interventions should be enhanced, alongside the current interventions in B-CoPCT, which tend to emphasize individual- and facility-level determinants including behavior change, provision of needles/syringes and medically assisted therapy, and management of co-morbidities.

Structural factors such as lack of employment and gender inequity mediate HIV risks among PWID by enhancing their vulnerabilities to HIV [30, 31, 43, 44]. Therefore, addressing structural determinants will be essential. Evidence supports our assertions on the importance of macro-level determinants of HIV infection. In a large study of 1633 Canadian PWID, structural factors such as having personal or social support, a regular place to stay, and formal employment opportunities all reduced risky drug injection behaviors [44]. In the United States, microfinance interventions and support with legitimate employment reduced the amounts and frequency of drugs that women used [45]. In Indonesia, females who injected drugs who were financially independent had more control on their drug use and attendant HIV risks [46]. Therefore, proactively combating homelessness, enhancing employment opportunities, and addressing gender-based economic inequity could mitigate the risk of HIV acquisition among PWID in Cambodia. Such approaches need to be explored as a policy priority.

### Study limitations

This study has some limitations. First, the cross-sectional study provides a snapshot of prevalence, which does not allow us to examine the temporality of the associations. Second, recruitment bias may exists because this study included 12 provinces with the highest burden of HIV and drug use, leaving out 13 provinces with lower burden of these phenomena, and a significant proportion of PWID was recruited from the capital city. However, our aim in recruiting a large sample from these high-burden sites for this national survey was to ensure the widest possible generalizability while ensuring that the study was feasible. Furthermore, any PWID identified in the provinces was included in the study. Given these complex sample survey data, the statistical methods such as Taylor series approximations should have been performed to adjust the standard errors for the sample design effects. Third, this study utilized self-reporting measures to gather sensitive information on drug use and sexual behaviors, which could have been affected by social desirability bias as is the case in other studies of PWID [47]. Finally, it is possible that the incentive provided to the participants may have affected their genuine motivation to participate, but we anticipate that the impact of the bias is minimal.

## Conclusions

This national survey found that the prevalence of HIV among PWID was 15.2%, which is a reduction from the 24.8% reported in a similar IBBS in 2012 [3, 5]. Among those currently infected with HIV, 57.4% were found to be co-infected with HCV. The prevalence of HIV was independently associated with being female, older, being widowed/divorced/separated, low level of formal education, low income, living on the streets, and having HCV infection. Given the current wide coverage of all the comprehensive package of HIV prevention, treatment, and care services in Cambodia [1, 2], we suggest that to prevent and control the HIV epidemic in this context, intervention programs and messages should be tailored to PWID with the above social demographic profiles, emphasizing on macro-structural interventions. Given that 44.7% of PWID in this study were not aware of their HIV status prior to the survey, it is important to reach the more hidden PWID through innovative strategies, and RDS method can be an effective approach to reach those who have not been reached by the traditional approaches.

## Data Availability

Data used for this study can be accessed upon request from the Principal Investigator (Dr. Siyan Yi) at ephsyi@nus.edu.sg

## Abbreviations

AIDS: Acquired immune deficiency syndrome
AOR: Adjusted odd ratio
B-CoPCT: Boosted Continuum of Prevention, Care, and Treatment
HCV: Hepatitis C virus
HIV: Human immunodeficiency virus
IBBS: Integrated biological and behavioral survey
IQR: Interquartile range
NECHR: National Ethics Committee for Health Research
NGO: Non-governmental organization
PWID: People who inject drugs
PWUD: People who use drugs
RDS: Respondent driven sampling
SD: Standard deviation
STI: Sexually transmitted infections

## References

[1] National AIDS Authority. Cambodia Country Progress Report (2015). Monitoring Progress Towards the 2011 UN political declaration on HIV and AIDS.

[2] Vun MC, Fujita M, Rathavy T, Eang MT, Sopheap S, Sovannarith S (2014). Achieving universal access and moving towards elimination of new HIV infections in Cambodia. J Int AIDS Soc.

[3] Chhea C, Heng S, Tuot S (2014). National Popualtion size estimation, HIV related risk behaviors, HIV prevalence among people who use drugs in Cambodia in 2012.

[4] Mburu G, Ngin C, Tuot S, Chhoun P, Pal K, Yi S (2017). Patterns of HIV testing, drug use, and sexual behaviors in people who use drugs: findings from a community-based outreach program in Phnom Penh. Cambodia Addict Sci Clin Pract.

[5] Sopheab H, Chhea C, Tuot S, Muir JA (2018). HIV prevalence, related risk behaviors, and correlates of HIV infection among people who use drugs in Cambodia. BMC Infect Dis.

[6] National Center for HIV/AIDS, Dermatology and STD. Standard Operation Procedure (SoP) for Boosted Continuum of Prevention to Care and Treatment for Most at Risk Population in Cambodia. Phnom Penh: National Center for HIV/AIDS, Dermatology and STD; 2013.

[7] Weissman A, Ngak S, Srean C, Sansothy N, Mills S, Ferradini L (2016). HIV prevalence and risks associated with HIV infection among transgender individuals in Cambodia. PLoS One.

[8] Granich R, Gupta S, Hall I, Aberle-Grasse J, Hader S, Mermin J (2017). Status and methodology of publicly available national HIV care continua and 90-90-90 targets: a systematic review. PLoS Med.

[9] Levi J, Raymond A, Pozniak A, Vernazza P, Kohler P, Hill A (2016). Can the UNAIDS 90-90-90 target be achieved? A systematic analysis of national HIV treatment cascades. BMJ Glob Health.

[10] Staveteig S, Croft TN, Kampa KT, Head SK (2017). Reaching the 'first 90′: gaps in coverage of HIV testing among people living with HIV in 16 African countries. PLoS One.

[11] Risher K, Mayer KH, Beyrer C (2015). HIV treatment cascade in MSM, people who inject drugs, and sex workers. Curr Opin HIV AIDS.

[12] World Health Organization (2009). Technical guide for countries to set targets for universal access to HIV prevention, treatment and Care for Injecting Drug Users.

[13] Tavitian-Exley I, Vickerman P, Bastos FI, Boily MC (2015). Influence of different drugs on HIV risk in people who inject: systematic review and meta-analysis. Addiction..

[14] United Nations High Commissioner for Human Rights (1996). Law on control of drugs.

[15] KHANA (2017). HIV/AIDS flagship annual Progress report to President's emergency plan for AIDS relief (PEPFAR)/United States Agency for International Development (USAID), Cambodia.

[16] White RG, Hakim AJ, Salganik MJ, Spiller MW, Johnston LG, Kerr L (2015). Strengthening the reporting of observational studies in epidemiology for respondent-driven sampling studies:“STROBE-RDS” statement. J Clin Epidemiol.

[17] Heckathorn DD (1997). Respondent-driven sampling: a new approach to the study of hidden populations. Soc Problems.

[18] Mun P, Chhim S, Chhoun P, Tuot S, Ly C, Dionisio J (2016). National population size estimation, HIV related risk behaviors, and HIV prevalence among men who have sex with men in Cambodia in 2014.

[19] Mun P, Tuot S, Chhim S, Chhoun P, Ly C, Pal K (2016). Integrated biological and behavioral survey among transgender women in Cambodia.

[20] Yi S, Chhoun P, Brant S, Kita K, Tuot S (2014). The sustainable action against HIV and AIDS in communities (SAHACOM): end-of-project evaluation.

[21] National Institute of Statistics (2015). Cambodia demographic and health survey 2014.

[22] Bell BA, Onwuegbuzie AJ, Ferron JM, Jiao QG, Hibbard ST, Kromrey JD (2012). Use of design effects and sample weights in complex health survey data: a review of published articles using data from 3 commonly used adolescent health surveys. Am J Public Health.

[23] Chhorvann C, Vonthanak S (2011). Estimations and projections of HIV/AIDS in Cambodia 2010–2015.

[24] Eluwa GI, Strathdee SA, Adebayo SB, Ahonsi B, Adebajo SB (2013). A profile on HIV prevalence and risk behaviors among injecting drug users in Nigeria: should we be alarmed?. Drug Alcohol Depend.

[25] Scheibe A, Makapela D, Brown B, dos Santos M, Hariga F, Virk H (2016). HIV prevalence and risk among people who inject drugs in five south African cities. Int J Drug Policy..

[26] Higgs P, Owada K, Hellard M, Power R, Maher L (2008). Gender, culture and harm: an exploratory study of female heroin users of Vietnamese ethnicity. Cult Health Sex.

[27] Evans JL, Hahn JA, Page-Shafer K, Lum PJ, Stein ES, Davidson PJ (2003). Gender differences in sexual and injection risk behavior among active young injection drug users in San Francisco (the UFO study). J Urban Health.

[28] Tuot S, Mburu G, Mun P, Chhoun P, Chann N, Prem K (2019). Prevalence and correlates of HIV infection among people who use drugs in Cambodia: a cross-sectional survey using respondent driven sampling method. BMC Infect Dis.

[29] Darke S (2011). The life of the heroin user: typical beginnings, trajectories and outcomes.

[30] Feng C, DeBeck K, Kerr T, Mathias S, Montaner J, Wood E (2013). Homelessness independently predicts injection drug use initiation among street-involved youth in a Canadian setting. J Adolesc Health.

[31] Whittaker E, Swift W, Roxburgh A, Dietze P, Cogger S, Bruno R (2015). Multiply disadvantaged: health and service utilisation factors faced by homeless injecting drug consumers in Australia. Drug Alcohol Rev.

[32] Crofts N, Louie R, Rosenthal D, Jolley D (1996). The first hit: circumstances surrounding initiation into injecting. Addiction..

[33] Hatzakis A, Sypsa V, Paraskevis D, Nikolopoulos G, Tsiara C, Micha K (2015). Design and baseline findings of a large-scale rapid response to an HIV outbreak in people who inject drugs in Athens. Greece: the ARISTOTLE programme Addiction.

[34] Razani N, Mohraz M, Kheirandish P, Malekinejad M, Malekafzali H, Mokri A (2007). HIV risk behavior among injection drug users in Tehran. Iran Addiction.

[35] Appel PW, Ellison AA, Jansky HK, Oldak R (2004). Barriers to enrollment in drug abuse treatment and suggestions for reducing them: opinions of drug injecting street outreach clients and other system stakeholders. Am J Drug Alcohol Abuse.

[36] Aceijas C, Rhodes T (2007). Global estimates of prevalence of HCV infection among injecting drug users. Int J Drug Policy.

[37] MacArthur GJ, van Velzen E, Palmateer N, Kimber J, Pharris A, Hope V (2014). Interventions to prevent HIV and hepatitis C in people who inject drugs: a review of reviews to assess evidence of effectiveness. Int J Drug Policy..

[38] van Griensven J, Phirum L, Choun K, Thai S, De Weggheleire A, Lynen L (2014). Hepatitis B and C co-infection among HIV-infected adults while on antiretroviral treatment: long-term survival, CD4 cell count recovery and antiretroviral toxicity in Cambodia. PLoS One.

[39] De Weggheleire A, An S, De Baetselier I, Soeung P, Keath H, So V (2017). A cross-sectional study of hepatitis C among people living with HIV in Cambodia: prevalence, risk factors, and potential for targeted screening. PLoS One.

[40] Lerolle N, Limsreng S, Fournier-Nicolle I, Ly S, Nouhin J, Guillard B, et al. High frequency of advanced hepatic disease among HIV/HCV co-infected patients in Cambodia: the HEPACAM study (ANRS 12267). J AIDS Clin Res. 2012;3(6).

[41] Chhim S, Ngin C, Chhoun P, Tuot S, Ly C, Mun P (2017). HIV prevalence and factors associated with HIV infection among transgender women in Cambodia: results from a national integrated biological and behavioral survey. BMJ Open.

[42] Rhodes T, Singer M, Bourgois P, Friedman SR, Strathdee SA (2005). The social structural production of HIV risk among injecting drug users. Soc Sci Med.

[43] Mburu G, Limmer M, Holland P (2019). HIV risk behaviours among women who inject drugs in coastal Kenya: findings from secondary analysis of qualitative data. Harm Reduct J.

[44] Luchenski S, Ti L, Hayashi K, Dong H, Wood E, Kerr T (2016). Protective factors associated with short-term cessation of injection drug use among a Canadian cohort of people who inject drugs. Drug Alcohol Rev..

[45] Sherman SG, German D, Cheng Y, Marks M, Bailey-Kloche M (2006). The evaluation of the JEWEL project: an innovative economic enhancement and HIV prevention intervention study targeting drug using women involved in prostitution. AIDS Care.

[46] Lazuardi E, Worth H, Saktiawati AM, Spooner C, Padmawati R, Subronto Y (2012). Boyfriends and injecting: the role of intimate male partners in the life of women who inject drugs in Central Java. Cult Health Sex..

[47] Latkin CA, Vlahov D, Anthony JC (1993). Socially desirable responding and self-reported HIV infection risk behaviors among intravenous drug users. Addiction..

